# Bibliotherapy

**DOI:** 10.5195/jmla.2019.696

**Published:** 2019-07-01

**Authors:** Eleanor Shanklin Truex

**Affiliations:** Clinical Librarian, Medical Library, AMITA Health Saint Joseph Hospital, Chicago, IL, and AMITA Health Saint Francis Hospital, Evanston, IL, etruex62@gmail.com

Like most librarians, I have always practiced an individual form of bibliotherapy, as defined in the introduction: “guidance and solace found through reading” (p. xiii). Intrigued by the premise that it could be formalized into a therapeutic modality, I was not disappointed with this book.

*Bibliotherapy* is structured with eleven contributors, two of whom are its editors. The flow is logical and coherent, starting with defining the field and moving through its history, theories, and trends, all covered ably by the two editors. The book’s second half presents case studies using various methods of bibliotherapy by diverse contributors. The case studies cover a range of situations: wellness, substance abuse, inpatient psychiatric wards, dementia, and even those at risk for homelessness. The cases are interesting, are pertinent to almost anyone in the library sciences (regardless of specific field), and feature common sense approaches to the myriad of scenarios that librarians find themselves in today. This is not a book designed solely for medical librarians, and that is one of its strengths.

The editors are to be lauded for pulling together a resource that provides such comprehensive and forthright information on the current status of bibliotherapy, its pitfalls, and its problems, as well as suggestions on how to strengthen it as a therapy. At the crux is this question: “In particular, the position of bibliotherapy at the edge of medical practice—was it a psychological therapy, a treatment, or a reading intervention?” (p. 8).

Some quantitative research has been done regarding bibliotherapy, with mixed results, which underscores that randomized controlled trials (RCTs) might not be the most appropriate method to study something as complex as bibliotherapy:

Meta-analyses which draw together RCTs to pool effect size and draw more significant conclusions showed that there were statistically significant positive effects on the participants in RCTs of bibliotherapy. (p. 11)

Bibliotherapy lends itself to qualitative research, and there has been success found via that type of research. The editors are clear that more research is needed to broaden the evidence base, but bibliotherapy has been shown to be effective. This book tackles all that and more, in clear, concise prose. Readers feel like they are following a lecture, but more of a TED Talk than an academic discourse. Do not be fooled by its friendly demeanor, McNicol and Brewster bring their documentation. This book is well-referenced, with each chapter or section having its own bibliography attached to it.

What I find most exciting is the movement of bibliotherapy out of the self-help book section and into literature and poetry. It is a formidable challenge, to be sure. As with all library science initiatives, the developers must ensure that they are meeting the needs of their proposed audience, with the caveat that those needs can change very quickly. Inclusivity and cultural sensitivity are musts. The purpose of a bibliotherapy program must be delineated clearly, but its material is open to interpretation at all levels. Nonjudgmental guidance is key, but so also is maintaining group or program cohesion. A fine line, but what an exciting one!

This book provides a thorough background along with real-life examples of piloted programs and national initiatives to show the reader the possibilities of bibliotherapy. The approaches are as varied as the libraries and librarians who provide it. I look forward to (hopefully) future editions that include bibliotherapy programs from regions that have not yet been represented.

The section on “Narrative Medicine” speaks to a different approach to perceiving patients using bibliotherapy. This is an idealized scenario, fraught with difficulties in today’s complex health care systems, but it is worth striving toward:

Nevertheless, healthcare does not find narratives easy to deal with. Medicine likes to deal with definites and diagnosis, so positioning the diverse, contradictory stories of patients within this field is difficult. (p. 52)

Yes, but only if the narrative is allowed to supplant the realities of the plan of care. The nursing profession already emphasizes holistic approaches to patient care. Bibliotherapeutic collaboration between nurses and librarians would be both refreshing and intriguing.

In terms of similar books, Pardeck’s *Using Books in Clinical Social Work Practice: A Guide to Bibliotherapy* (Haworth Press; 2013) provides some background on the history of bibliotherapy, but it has a directly clinical focus. The two would complement one another.

McNicol and Brewster’s *Bibliotherapy* is of use in any library, even a small hospital library, if only so that the collection has a reference that is on trend. Its contents clearly exhibit the at-times obscure line from research to practice and affirm the importance of library services.

